# The evolving field of nephrology: what comes next? A report from the European Renal Association Scientific Advisory Board

**DOI:** 10.1093/ckj/sfag136

**Published:** 2026-04-28

**Authors:** Mehmet Kanbay, Sidar Copur, Giovambattista Capasso, Kultigin Turkmen, Pierre Delanaye, Annette Bruchfeld, Samar Abd ElHafeez, Charles J Ferro, Marianne C Verhaar, Francesco Pesce, Vassilios Liakopoulos, Peter Boor, Marcin Adamczak, Marina Vivarelli, Juan F Navarro-González, Rafael Kramann, Sandrine Florquin, Jose Manuel Valdieso

**Affiliations:** Department of Internal Medicine, Division of Nephrology, Koc University School of Medicine, Turkey; Department of Internal Medicine, Koc University School of Medicine, Turkey; Biogem, Institute of Molecular Biology and Genetics, Italy; Department of Internal Medicine Division of Nephrology, Necmettin Erbakan University Faculty of Medicine, Turkey; Department of Nephrology, Dialysis, Transplantation, CHU Sart Tilman, University of Liège, Belgium; Department of Nephrology-Dialysis-Apheresis, Hôpital Universitaire Carémeau, Université de Montpellier, France; GIGA Metabolism and Cardiovascular Biology, University of Liège, Belgium; Department of Health and Caring Sciences, Linköping University, Sweden; Department of Renal Medicine, Karolinska University Hospital and CLINTEC Karolinska Institutet, Sweden; Department of Epidemiology, High Institute of Public Health, Alexandria University, Egypt; Department of Renal Medicine, University Hospitals Birmingham, and Institute of Cardiovascular Sciences, University of Birmingham, B15 2TT, UK; Department of Nephrology, University Medical Center Utrecht, Netherlands; Department of Translational Medicine and Surgery, Università Cattolica del Sacro Cuore, Italy; Division of Renal Medicine, Ospedale Isola Tiberina-Gemelli Isola, Italy; 2nd Department of Nephrology, AHEPA University Hospital Medical School, Aristotle University of Thessaloniki, 54124, Greece; Institute for Pathology, University Hospital Aachen, RWTH Aachen University, Germany; Department of Nephrology, Transplantation and Internal Medicine, Medical University of Silesia, Poland; Laboratory of Nephrology and Clinical Trial Center, Bambino Gesù Children’s Hospital IRCCS Rome, Italy; Facultativo Especialista Servicio de Nefrología, Hospital Universitario Nuestra Señora de Candelaria, Spain; Department of Medicine 2, RWTH Aachen University, Medical Faculty, Germany; Department of Internal Medicine, Nephrology and Transplantation, Erasmus Medical Center, The Netherlands; Department of Pathology, AmsterdamUMC location University of Amsterdam, Netherlands; Institut de Recerca Biomedica de Lleida - Fundacio Dr Pifarre, IRBLleida,, 25198, Spain; RICORS 2040, Instituto de Salud Carlos III, Spain

**Keywords:** biomarker, chronic kidney disease, nephrology, positron emission tomography, therapeutics

## Abstract

The field of nephrology is rapidly expanding and evolving with advancements in the areas of diagnostics including biomarkers, molecular genetics and imaging modalities, and therapeutics. As acute kidney injury affects a substantial proportion of hospitalized patients with considerable morbidity and mortality risk and chronic kidney disease is among the leading causes of morbidity and mortality globally, the advancements in the field of nephrology are worth focusing on and highlighting. Novel biomarkers along with better imaging modalities, including functional tools such as positron emission tomography and contrast-enhanced ultrasound, may enable earlier diagnosis of kidney injury and potentially reverse injury state before the establishment of an irreversible state of injury. Moreover, there are multiple ongoing clinical trials evaluating novel therapeutic approaches, advancements in kidney replacement therapies or targeted approaches against kidney transplantation including xenotransplantation and plasma cell-directed therapies. In addition, artificial intelligence may become a game changer in nephrology, providing improved diagnostic and therapeutic alternatives. However, the limitations of current state of knowledge in those novel modalities should be adequately acknowledged and addressed. In this narrative review, our aim is to evaluate the current state of advancements in the field of nephrology in terms of diagnostics, imaging modalities, biomarkers, and therapeutics along with providing potential future perspectives.

## INTRODUCTION

Chronic kidney disease (CKD), a condition characterized by irreversible and potentially progressive alteration of kidney function, is a leading cause of morbidity and mortality globally by affecting >850 million people surpassing diabetes mellitus and chronic obstructive pulmonary disease [1, 2]. The devastating complications of CKD including higher cardiovascular and cerebrovascular disease risk, cognitive decline, bone-mineral disorders, hypertension, and all-cause mortality necessitate the need for improvements in the diagnostics and therapeutics. Epidemiological studies have revealed a 1.19% increase in average annual percentage change in prevalence accompanied by a significant increase in mortality and disability-adjusted life-years lost [3–5]. As the prevalence of CKD is expected to rise in the upcoming decades along with CKD-associated comorbidities and mortality, such an issue requires careful and extensive workup. We hereby aim to review the latest advancements in the field of nephrology in terms of early biomarkers, diagnostic approaches, therapeutics, and kidney replacement therapies including novel transplantation strategies. We further discuss their future applications and their potential to change the practice of nephrology in the near and far future. Finally, we provide a commentary on the potential role of artificial intelligence to change the landscape of nephrology as we currently understand it.

### Future perspectives for biomarkers

Serum creatinine and cystatin C measurements are highly employed serum biomarkers for renal function assessment, however, their sensitivity to detect early changes or injuries is highly limited by multiple confounding factors potentially interfering with their measurements [6]. Neutrophil gelatinase-associated lipocalin (NGAL), kidney injury molecule (KIM)-1, liver type fatty acid binding protein, urinary tissue inhibitor of metalloproteinases-2 (TIMP-2), insulin-like growth factor-binding protein-7, urinary Dickkopf-3, urinary monocyte chemoattractant protein-1, interleukin-18, and urinary uromodulin have appeared as promising alternatives over the past decade [6]. We hereby aim to review novel biomarkers that may aid in earlier diagnosis of kidney dysfunction and inform clinical management.

#### Calprotectin

Calprotectin, an innate immune system protein released from neutrophils and macrophages during an immune response, has been hypothesized to be an early indicator of kidney dysfunction. A cross-sectional study involving 86 acute kidney injury (AKI) patients and 15 healthy controls has illustrated the predictive power of urinary calprotectin levels in the differentiation of intrinsic AKI from pre-renal causes, as intrinsic AKI patients have 60.7 times higher urinary calprotectin levels [7]. Similarly, sensitivity and specificity of urinary calprotectin levels have shown to be 0.90 and 0.93, respectively, with a pooled diagnostic accuracy of 0.96 on differentiation of intrinsic AKI as evident from a recent meta-analysis study [8]. Even though the exact pathophysiological role of calprotectin in AKI is yet to be established, the release from tubular cells and neutrophilic infiltrations involved in intrinsic AKI appears to be the major source of urinary calprotectin [9]. The primary advantages of such a biomarker include non-invasive and reliable distinction of pre-renal versus intrinsic causes of AKI, which may completely shift the therapeutic approach for an individual patient. Moreover, it enables earlier detection of kidney injury, well before an increase in serum creatinine or cystatin C or even before an increase in NGAL [10]. Another major advantage is the reliable prediction of outcomes of AKI including risk for renal replacement therapy initiation and mortality [11].

A prospective cohort study involving a total of 4.662 patients without CKD at baseline has illustrated the predictive power of higher serum calprotectin levels in new-onset CKD [12]. On the other hand, urinary calprotectin fails to predict CKD progression on a prospective cohort study involving 143 diabetic and/or hypertensive nephropathy patients [13]. The crucial differences in this study are the exclusion of patients with an eGFR decline >5 ml/min/1.73 m^2^ in the last 12 months and inclusion of non-inflammatory etiologies of CKD. By including only patients with a stable estimated glomerular filtration rate (eGFR), the study sought to exclude those with active kidney injury, which might reflect an inflammatory process capable of confounding the interpretation of urinary calprotectin levels.

Urinary, and potentially serum, calprotectin measurements seem to have high and promising potential to be included in AKI workup in the following years, especially for the differentiation of intrinsic AKI cases. However, the current literature regarding the use of either urinary or serum calprotectin measurements in AKI and/or CKD patients is scarce and require further large-scale prospective clinical evidence before reaching widespread clinical applicability. Currently, calprotectin is not included in any major clinical guidelines for kidney diseases. The role of calprotectin in CKD patients as biomarker of disease progression or complications such as vascular calcification [14] should be further examined in future clinical trials before reaching for a stricter interpretation.

#### Soluble urokinase-type plasminogen activator receptor

Soluble urokinase-type plasminogen activator receptor (suPAR), a GPI-anchored membrane-bound protein involved in many aspects of immune response including cellular adhesion, differentiation, proliferation, and migration, has emerged as a potential biomarker for kidney diseases. The idea behind a circulating plasma factor involved in AKI and CKD first arose from patients with focal segmental glomerulosclerosis and was later supported by the demonstration of elevated suPAR levels that lead to podocyte injury [15]. The interaction between elevated suPAR in such patients with podocytes is mediated via v3αvβ3 integrins on podocytes [16].

A 2023 meta-analysis conducted on nine clinical studies has revealed statistically significantly higher levels of suPAR among AKI patients compared with healthy controls (5.23 ± 4.07 vs. 3.23 ± 0.67 ng/mL, *P* < .001). Moreover, sensitivity and specificity of suPAR have shown to be 0.77 and 0.64, respectively, in the prediction of AKI as illustrated by a meta-analysis study by Huang and colleagues in of 2319 patients [17]. The superiority of suPAR over traditional biomarkers of renal function, creatinine, and urea is associated with an earlier rise after kidney insult allowing for earlier detection and diagnosis, not being affected by age, gender, muscle mass, or nutrition [18]. Moreover, elevated suPAR levels can reliably predict future eGFR decline in patients presenting with renal injury yet with normal eGFR [19]. Furthermore, suPAR levels have shown to be elevated among CKD patients, illustrated by another meta-analysis study involving 14 clinical studies. In addition, elevated suPAR levels independently raise the risk for mortality (*P* = .001), cardiovascular disease (*P* < .001), and end-stage kidney disease (ESKD) (*P* < .001) [20].

To conclude, suPAR appears to be a valid predictor of AKI and CKD that may become clinically relevant in the coming years despite currently not being included in any major nephrology guidelines. An important aspect to consider when evaluating suPAR levels in clinical practice is that it is not only an indicator of kidney injury, but it is also involved in disease pathogenesis. Few clinical trials have revealed that suPAR levels decline with various treatment options including cyclosporine, corticosteroids, rituximab, finerenone, and sodium-glucose cotransporter-2 inhibitor, which is well correlated with disease remission (NCT01468493, NCT01573533, NCT01847092, NCT04782245). However, clinical evidence on the use of suPAR levels as a marker of treatment follow-up or treatment response evaluation is currently hypothetical and at proof-of-concept state.

#### CXCL9

C-X-C motif ligand (CXCL)-9, a chemokine induced by interferon-gamma that upregulates T lymphocytes, has been proposed as a reliable indicator of AKI, especially interstitial nephritis. With the widespread use of immune-checkpoint inhibitor (ICI) therapy in the field of medical oncology, the recognition of adverse effects is at most importance as it leads to core treatment decisions. A proteomics-based clinical study of 79 patients receiving ICI therapy has revealed that higher urinary CXCL9 levels are the best predictor of ICI-induced acute interstitial nephritis compared with other biomarkers [21]. Moreover, higher levels of urinary CXCL9 among ICI-treated patients with interstitial nephritis have also been reported in other clinical studies [22, 23]. Similarly, a high positive predictive value of urinary CXCL9 has also been established in another cohort study utilizing proteomics analysis among patients without solid organ malignancies and ICI therapy [24]. However, the major limitation of such a biomarker is the lack of specificity as most oncological patients experience a pro-inflammatory state leading to higher baseline CXCL9 levels. Therefore, there is a clear need for future clinical studies evaluating the levels of such a biomarker among patients receiving other anti-neoplastic agents or experiencing non-interstitial nephritis-related AKI.

Furthermore, CXCL9 has been investigated as a potential early biomarker for allograft dysfunction and prediction of long-term graft outcomes among kidney transplant recipients. A recent prospective cohort study of 622 kidney transplant recipients has demonstrated the high predictive power of CXCL9 when combined with clinical data and CXCL10 measurement in terms of acute rejection episodes [25]. Although this study did not provide longitudinal data on the progression of CXCL9 levels, it is still crucial step as it appeared to be a potential predictor of allograft dysfunction. Another large-scale clinical study of 280 adult and pediatric kidney transplant recipients has revealed that low urinary CXCL9 levels in the context of allograft dysfunction may be utilized to rule out inflammatory conditions including infectious etiologies and rejection episodes. Moreover, absent urinary CXCL9 levels at post-transplant month 6 may be a valid indicator of low future immunological risk for the recipient [26]. Therefore, CXCL9 appears to provide early clinical promise as a biomarker for inflammatory kidney disorders on native or transplant kidneys. Unfortunately, the primary area of utilization may be restricted to acute interstitial nephritis and acute rejection episodes, however, such an understanding is subject to alterations in future clinical studies.

### Future perspectives for kidney imaging

The evaluation of AKI or CKD involves combination of history, physical examination, imaging modalities, laboratory workup, and, in some cases, kidney biopsy. Nonetheless, currently available diagnostic imaging modalities including ultrasonography, computed tomography, or magnetic resonance imaging are highly limited to the detection of post-renal obstructive pathologies with only limited diagnostic power in early diagnosis as most anatomical alterations evident on such modalities do not evolve until a late disease stage. Multiple novel imaging modalities are being investigated with the aim of detecting and diagnosing kidney disease at an earlier stage, and potentially guiding follow-up and treatment [27].

#### Positron emission tomography-computed tomography

Positron emission tomography-computed tomography (PET-CT), a multidimensional imaging modality combining anatomical data from CT and functional data from PET modalities that reflects glucose uptake and tissue metabolism and is primarily used in tumor staging and oncological treatment response assessment [28], has recently been investigated in the diagnosis and follow-up of benign conditions. Such a potential role has also been investigated in the field of nephrology. The demonstration by Katagiri and colleagues of higher fluorodeoxyglucose (FDG) uptake at renal cortex among patients with ICI-induced interstitial nephritis with a non-oliguric state or hemodialyzed state has pioneered the field of PET-CT as an imaging modality in kidney diseases [29].

Infectious diseases of the kidney including acute and/or chronic pyelonephritis, renal abscess, or cyst infection, especially among autosomal dominant polycystic kidney disease patients, appear to be the strongest indication for PET-CT imaging in the field of nephrology. The sensitivity and specificity of FDG-PET-CT in the diagnosis of acute complicated pyelonephritis have been shown to be 100% and 90%, respectively, along with focal FDG uptakes correlating with the abscess formation requiring drainage [30]. Similarly, a single-center study with a total of 30 ADPKD patients has revealed sensitivity of 88.9% and specificity of 75.0% for FDG-PET-CT for cyst infection [31]. Such a diagnostic approach is based on the knowledge that uncomplicated cysts in ADPKD are fluid filled sacs with no FDG uptake with FDG uptake indicating inflammation and/or infectious process. Even though data are premature to shed light on a clear pathway, FDG-PET may have a fair chance of becoming a part of the diagnostic algorithm for complicated urinary tract infections, especially for difficult-to-diagnose challenging cases. The 68Ga-fibroblast activation protein inhibitor (FAPI) PET-CT is a promising alternative to FDG-PET-CT in which FAPI is a quinoline-based membrane-bound protein glycoprotein enzyme only expressed in conditions with either malignancies or inflammation [32]. However, the effectiveness of such a radiotracer in acute pyelonephritis has yet to be confirmed by future clinical trials as the current evidence is based on case reports [33].

The knowledge of the role of PET-CT in the diagnosis or follow-up of primary glomerulonephritis patients is highly limited to case reports and case series [34–36]. There is a clear need for future large-scale clinical trials to evaluate the role of PET-CT in such cases, however, such studies are too premature to reach for a definitive conclusion. However, such an association may most likely require considerable effort when considering significant heterogeneity of primary glomerulonephritis in terms of underlying pathophysiology and patient characteristics, therapeutic regimens, and disease course. A study using 18F-FDG-PET/CT found diffuse renal 18F-FDG uptake to be associated with the presence of AKI, and this uptake was reversible with renal function improvement [37].

CKD, characterized by irreversible renal fibrosis of varying degrees, has been another area of interest in PET-CT research. A recent small study involving a total of 13 CKD patients who underwent both kidney biopsy and 68Ga-FAPI PET-CT to assess renal fibrosis demonstrated the effectiveness of this imaging modality. The results showed significantly higher SUVmax values in patients with grade II/III renal fibrosis compared with healthy individuals. However, the low number of participants with unequal number of patients at each fibrosis stage and lack of data on future predictive value of FAPI scan are the major limitations of this cross-sectional study [38]. Another clinical trial involving a total of 81 patients investigated the association of 68Ga-FAPI, 68Ga-prostate-specific membrane antigen, or 68Ga-DOTA-Tyr3-octreotide on predicting fibrosis stage. A statistically significant negative correlation between renal parenchymal 68Ga-FAPI uptake and eGFR without any such association with other tracers has been revealed. Nonetheless, an unequal number of patients at each CKD stage or tracer groups along with inclusion of patients with disseminated malignancies that may interfere with tracer distribution are the primary limiting factors [39]. Moreover, the validity of Ga-FAPI scan has been investigated on 29 lupus nephritis patients undergoing kidney biopsy within 1 week of a Ga-FAPI scan. Renal Ga-FAPI uptake has shown to correlate with serum creatinine levels, renal tubulointerstitial fibrosis levels, and chronicity index on biopsy materials [40]. Also, there are significant discrepancies in terms of areas for background activity assessment, including skeletal muscle, lung parenchyma, and myocardium, which are important concerns necessitating future clinical studies.

The role of PET-CT in the evaluation of rejection episodes among kidney transplant recipients is another area of research with significant hope. A single-center observational study involving 31 kidney transplant recipients has illustrated the strong negative predictive power of FDG-PET-CT in subclinical and clinical rejection episodes along with a positive correlation of SUVmax measurements with the composite Banff scores of the histopathological evaluation [41]. A similar study could confirm this finding [42]. Another trial has demonstrated the high negative predictive value of negative FDG-PET-CT combined with urinary CXCL-9/creatinine ratios in the exclusion of rejection episodes of 91 kidney transplant recipients [43]. Therefore, FDG-PET-CT could be a valid and efficient methodology to exclude rejection episodes among kidney transplant recipients presenting with reduction of eGFR. However, the sensitivity of PET-CT for positively predicting rejection episodes or discrimination rejection episodes from infectious or malignant causes is poor.

#### FDG-PET-magnetic resonance imaging

FDG-magnetic resonance imaging (FDG-MRI), an integrative model combining MRI and PET, has been evaluated as a non-invasive imaging modality to assess eGFR and effective renal plasma flow. A proof-of-concept study on 24 healthy participants has confirmed the highly correlated estimation of eGFR and effective renal plasma flow with FDG-PET-MRI [44]. Similarly, another clinical study in which 24 healthy participants underwent simultaneous FDG-PET and MRI has confirmed that split function, mean transit time, and outflow efficiency are significantly correlated with their reference values assessed via MAG3-sintigraphy [45]. Another promising and exiting area of research in the applicability of FDG-PET-MRI is the prediction of AKI resolution among kidney transplant recipients. A cohort study involving 13 kidney transplant recipients experiencing AKI episodes and 24 healthy controls undergoing an FDG-PET-MRI scan has demonstrated the statistically significant predictive power of initial flow on AKI resolution and renal response power on tubular damage [46].

The data regarding the use of FDG-PET, either integrated with a CT or MRI scan, on various kidney diseases is not only intriguing and promising but also has offered early clinical promise. Such an approach may hypothetically eliminate the need for diagnostic invasive procedures and associated risks as evident by the data indicating high negative predictive power on allograft rejection, even the subclinical episodes. The fundamental superiority of PET scan in the evaluation of renal disorders is the integration of functional assessment to anatomical context while functional workup is not only limited to malignant process but includes inflammatory processes that are the key pathophysiological mechanisms in almost all disorders. However, the distinctive power of a PET scan for various benign inflammatory conditions is unfortunately poor. Therefore, at present, a PET scan may aid in the selection of patients for further diagnostic workups such as biopsy but not directly point to a specific disorder without further workup. However, such an understanding may be subject to evolution with future large-scale clinical trials in various patient groups.

#### Contrast-enhanced ultrasound

Contrast-enhanced ultrasonography (USG), utilizing an intravenous microbubble contrast material coated with lipid/phospholipid shells, is a novel imaging modality implication in renal mass characterization including the Bosniak classification of renal cysts but is also used for assessment of kidney transplant complications, acute pyelonephritis, renal infarction, and many other aspects such as the measurement of the renal vasculature [47]. The primary advantages of such a modality include no risk for nephrotoxicity as it is excreted via the lungs and destroyed via ultrasound waves; in addition, there is no exposure to radiation.

The effectiveness of contrast-enhanced USG on the evaluation of AKI has been evaluated in a clinical study involving sepsis-associated AKI patients and control groups with a statistically significant decline in the renal blood flow and time-averaged velocity among AKI patients (*P* < .02 and .001, respectively). Moreover, aggravation of AKI status led to an even greater decline in renal blood flow [48]. The clinical application of contrast-enhanced USG on AKI patients has been evaluated in a few other clinical trials [49, 50]. However, most of those trials have undeniable limitations disabling strong recommendations on the use of contrast-enhanced USG in clinical practice of AKI evaluation including: (i) a considerably low number of participants with significant heterogeneity; (ii) short follow-up periods disabling prediction of long-term prognostic data; (iii) a mostly single-center observational study design; (iv) a lack of confirmatory histopathological examination of all participants; and (v) a low number of high-risk patients including AKI patients requiring renal replacement therapies.

Certain parameters evaluated on contrast-enhanced USG have shown to be an important predictor of CKD status and/or CKD progression. A prospective cohort study involving 167 patients with a median follow-up period of 30.4 months has revealed that lower derived peak intensity on USG has a significant association with CKD progression evident after regression analysis with an additional significant association with renal chronicity score on biopsy specimens [51]. In addition, such associations were also evident on few other clinical trials [52, 53]. A more significant and potentially groundbreaking application of such an imaging modality is the prediction of AKI-to-CKD transition. Promising data have come from the demonstration of a significant association of renal perfusion assessment 1–21 days after an ischemic injury with later renal functional impairment and renal fibrosis. The renal perfusion is expected to return to baseline after a minimal ischemic insult with no long-term fibrotic effect, while inability to return to the baseline at day 1 after an ischemic insult predicts poorer long-term outcomes [54].

Furthermore, the half descending time (DT/2) parameter has shown to be a reliable indicator available to differentiate between rejection and non-rejection mediated allograft dysfunction with 76.5% sensitivity and 81.2% specificity. However, such a significant link has not been established in other USG parameters such as peak intensity or mean transit time [55]. Nevertheless, major limitations of this study, including lack of allograft biopsies in all participants, a single-center design with a relatively low number of kidney transplant recipients, and a lack of association with long-term prognostic data, should not be overlooked while interpreting the outcomes. Similar beneficial effects of contrast-enhanced USG on the evaluation of kidney transplant recipients have also been established in many other clinical trials [56, 57]. The American College of Radiology 2024 guidelines recommend use of contrast-enhanced USG in the assessment of allograft dysfunction to differentiate between rejection from non-rejection causes of AKI especially when eGFR is below 30 ml/min/1.73 m^2^ based on the data showing that such an imaging approach may reduce biopsy needs by 20%–40% [58]. On the other hand, the European Federation of Societies for Ultrasound in Medicine and Biology 2024 guidelines [59] and American Institute of Ultrasound in Medicine 2024 guidelines [60] limit the recommendation to use in early postoperative complications including vascular leak, ischemia, infarction, and acute tubular necrosis with strong evidence level.

To conclude, contrast-enhanced USG appears to be a potentially promising imaging modality with major advantages over other modalities such as lack of radiation exposure and nephrotoxicity risk, predictive power for AKI-to-CKD progression and/or CKD progression, and earlier detection of CKD-associated alterations at microvascular levels. Large-scale clinical trials with longer follow-up periods and a higher number of participants are essential before becoming a fundamental part of clinical practice, however, such a time does not appear to be far in the future.

#### MR elastography

MR elastography (MRE), a diagnostic modality assessing tissue stiffness and therefore fibrosis through shear wave diffusion caused by mechanical vibration, is a commonly employed technique to assess liver fibrosis [61], however, applicability to kidney parenchyma is still under investigation.

Evaluation of 16 kidney allograft recipients at least 1 year after transplant undergoing allograft biopsies with MRE has revealed a statistically significant positive correlation between MRE findings and biopsy-derived Banff fibrosis scores and a negative correlation between MRE and eGFR [62]. Similarly, another cross-sectional study with 25 CKD patients and five healthy controls has illustrated higher mean stiffness measurements on MRE among CKD patients compared with healthy controls along with a positive correlation with CKD stage [63]. However, contradictory results indicating lower renal stiffness measurements among CKD patients have also been reported among IgA nephropathy [64], diabetic nephropathy [65], and lupus nephritis [66] patients. Even though the exact underlying reason for such discrepancy and inconsistency is yet to be determined, the hypothesis includes heterogeneity of fibrotic kidney parenchyma leading to variations at MRE measurements, deep retroperitoneal location of the kidney interfering with MRE, and alterations at renal perfusion [67]. To conclude, MRE is a well-established and efficient tool for liver fibrosis evaluation but the state of the evidence in the assessment of kidney fibrosis is currently at the proof-of-concept stage at best.

#### Blood oxygenation level dependent MRI

Chronic hypoxia and impairment of tissue perfusion is the end-stage findings of CKD, while blood oxygenation level dependent (BOLD)-MRI aims to provide a non-invasive means to evaluate tissue oxygenation through the effective transverse relaxation time T2 parameter of MRI, which is expected to be shortened when deoxyhemoglobin levels rise [68]. Although BOLD-MRI is primarily designed to evaluate neuronal activity in the central nervous system, the potential applicability of such a model to renal oxygenation has been established in experimental animal models, mice and pigs, of AKI with validated changes recorded after application of loop diuretics [69–72]. Nonetheless, the results from CKD models are inconsistent in terms of decline in the effective transverse relaxation time T2 parameter. Even though there are few studies with a low number of participants potentially implicating decline in T2 among CKD patients [73, 74], contradictory findings currently predominate [75–77]. The inconsistent results may be attributable to the low numbers of participants with significant heterogeneity or variations of CKD etiologies. However, some other factors also need to be considered: first, deoxyhemoglobin level is not the only predictor of tissue T2 as it may be affected by the presence of inflammatory cells and tissue edema, hydration status, and renal perfusion [78]. Second, BOLD-MRI assesses intravascular deoxyhemoglobin levels, which are, under physiological circumstances, at equilibrium with extravascular levels but such a principle may not be applicable to fibrotic renal parenchyma in which microvascular obliteration may prevent such equilibrium. Therefore, there is a clear requirement for further clinical trials before widespread clinical use of BOLD-MRI as a non-invasive tool for renal fibrosis assessment.

### Future of kidney therapeutics

Along with the advancements in diagnostics and biomarkers in AKI and CKD, there have been many advancements in the field of therapeutics. Multiple approaches targeting: the renin–angiotensin–aldosterone system including siRNA-based modalities targeting angiotensinogen gene expression and non-steroidal mineralocorticoid receptor antagonists including finerenone; classic and alternative complement pathways including pegcetacoplan (C3), avacopan (C5a receptor), iptacopan (Factor B), endothelin receptors such as atrasentan, and dual renin–angiotensin and endothelin receptor blockers such as sparsentan and A Proliferation-Inducing Ligand (APRIL) inhibitors, have been employed in clinical trials with promising outcomes.

#### Soluble guanylate cyclase stimulators

The soluble guanylate cyclase-mediated nitric oxide-cyclic guanosine monophosphate pathway is impaired in CKD leading to impairment of NO production and tissue responsiveness to NO, ultimately causing endothelial dysfunction [79] Following the failure of praliciguat to produce a significant reduction in albuminuria in the Phase 2 clinical trial [80], soluble guanylate cyclase stimulators have regained research and clinical interest with the development of avenciguat. Avenciguat is a novel NO-independent activator of soluble guanylate cyclase aiming to restore the NO-cGMP pathway to improve renal and cardiovascular outcomes among CKD patients [81]. A demonstration with avenciguat showed a significant decline in albuminuria along with a relatively safe adverse effect profile evidenced in a Phase 1b randomized clinical trial conducted on diabetic CKD patients with a baseline eGFR of 20–75 ml/min/1.73 m^2^ and UACR between 200–3500 mg/g creatinine [82] has led to significant hope for future clinical trials. A pooled analysis of two randomized double-blind placebo-controlled clinical trials on CKD patients with a baseline eGFR of 20–90 ml/min/1.73 m^2^ and UACR of 200–3500 mg/g creatinine has recently been published by Heerspink and colleagues. A total of 500 patients undergoing placebo or avenciguat therapy (1, 2, or 3 mg three times daily dosage) over a 20-week period were included in the analysis. Placebo-corrected mean changes in albuminuria were −15.5% (95% CI −26.4 to −3.0) with 1 mg, −13.2% (95% CI −24.6 to −0.1) with 2 mg, and −21.5% (95% CI −31.7 to −9.8%) with 3 mg dosing. Avenciguat reduced albuminuria in patients with CKD irrespective of concomitant SGLT2i. Important considerations include a considerable decline in blood pressure, especially within 4 hours of drug administration reflecting acute vasodilatory effects that attenuate with time indicating tolerance to such effects. However, a lack of long-term follow-up data disabling interpretation of efficiency and safety over long-term use and drug adherence issues with three times daily dosing are the primary limitations of avenciguat therapy [83].

Gansevoort *et al*. completed a short-term randomized placebo-controlled study clinical study (8 weeks) with runcaciguat once daily, titrated weekly (30–120 mg if tolerated) (CONCORD). In this study, 243 patients with CKD and established atherosclerotic cardiovascular disease or heart failure, plus type 2 diabetes and/or hypertension, were enrolled. All patient received stable maximum tolerated ACEI or ARBs with or without SGLT2i. UACR decreased by −45.2% vs placebo with runcaciguat in patients with CKD without SGLT2i (*P* < .001) and by −48.1% versus placebo in patients with CKD treated with SGLT2i (*P* = .02) Therefore, runcaciguat, similar to the previously discussed avenciguat, reduced albuminuria in patients with CKD irrespective of concomitant SGLT2i. Both sGC activators were well tolerated. It important that these agents do not interfere with RAS and do not have the risk of hyperkalemia. Therefore, sGC activation may represent a novel kidney-protective treatment in CKD patients, which will be added to the top of the list of currently used pharmacological nephroprotection with ACEI or ARBs with SGLT2i [84].

#### Zilebesiran

A first-in-class siRNA-based therapeutic approach, namely zilebesiran, targeting and downregulating expression of the *AGT* gene encoding for angiotensinogen, has been under investigation for the treatment of hypertension [85]. Zilebesiran consists of a short, double-stranded RNA sequence composed of ∼20 pairs of nucleotides that are connected to *N*-acetylgalactosamine (GalNAc). The potential advantages of such an approach include: (i) suppression of the renin–angiotensin–aldosterone system at an earlier stage leading to broader inhibition of the system; (ii) the lack of “angiotensin II escape” mechanisms observed with angiotensin converting enzyme inhibitors or angiotensin receptor blocker therapy; (iii) conjugation of *N*-acetylgalactosamine to drug leading to liver-specific effects with none to minimal effects on extra-hepatic tissues limiting potential adverse effects; there is almost no risk for bradykinin pathway upregulation minimizing the risk of angioedema or other allergic effects [85]; (iv) the complete specificity of zilebesiran’s action, inhibiting only the expression of angiotensinogen, results from the unique sequence of siRNA bases only complementary to angiotensinogen mRNA; (v) the extremely long-lasting effect of zilebesiran is caused by the slow transfer of siRNA from endosomes and the possibility of the multiple degradation of angiotensinogen mRNA by the activated RISC-single-stranded; and (vi) single administration of this drug in a 6-month period allows for better blood pressure because it eliminates the daily need to take the drug, which should significantly reduce non-adherence [85, 86].

A randomized controlled double-blind placebo-controlled clinical trial involving a total of 377 patients with mild-to-moderate hypertension (135–160 mmHg systolic BP) after an antihypertensive drug washout period has revealed a statistically significant decline in blood pressure over 3 and 6 months of treatment assessed via 24-hour ambulatory blood pressure monitoring [87]. Moreover, zilebesiran therapy has shown a promising and statistically significant decline in blood pressure when used as an add-on therapy to patients with uncontrolled hypertension under indapamide, amlodipine, or olmesartan treatment according to a Phase 2 randomized placebo-controlled clinical trial of 1491 patients [88]. Such findings confirm the superior suppressive effects of zilebesiran indicated by significant blood pressure decline, even among patients already receiving olmesartan therapy. To conclude, zilebesiran therapy appears to be an effective and safe therapeutic alternative even when used as monotherapy or as an add-on therapy to other pharmacotherapies. However, such a novel therapeutic alternative has not yet received approval by any major organizations or been included in any clinical guidelines. Zilebesiran reduces blood pressure slowly, therefore it is not used in the treatment of hypertensive crises, but because of the gradual reduction in blood pressure, hypotensive episodes are not observed immediately after drug administration. Drug tolerance in the patients studied was good. The most common adverse effects were a transient, local, mild inflammatory skin reaction at the injection site and hyperkalemia. It is worth noting that hyperkalemia was mild, did not lead to termination of participation in the study, and involved 5% of the study participants [86]. However, it should be noted that the still unpublished results of the recent Phase 2 (KARDIA-3) trial completed among the patients with difficult-to-treat hypertension are disappointing. A single dose of zilebesiran 300 mg led to a 5-mmHg reduction in SBP at 3 months compared with the placebo, a difference that did not reach statistical significance. The true role zilebesiran in antihypertensive treatment (also in CKD patients) will be known after the completion of Phase 3 outcomes trial in patients with uncontrolled hypertension i.e. ZENITH (ZilebEsiraN Cardiovascular Outcome Study in Hypertension) trial. ZENITH will be a Cardiovascular Outcome Trial (CVOT) enrolling ∼11 000 patients and evaluating zilebesiran (300 mg) every 6 months compared with placebo in patients with uncontrolled hypertension on two or more antihypertensive drugs (one of them being a diuretic).

Other siRNA-based therapeutic alternatives are being investigated in various kidney diseases. Cemdisiran, aiming to downregulate hepatic expression of complement component 5 to inhibit membrane attack complex formation and function, is a novel therapeutic approach for patients with IgA nephropathy [89]. Cemdisiran therapy has led to rapid and prominent decline in C5 production as evidenced in a Phase 1 trial [90]. A double-blind Phase 2 clinical trial involving a total of 31 patients with IgA nephropathy and urinary protein excretion ≥1 g/24 hours has illustrated a 37.4% decline in proteinuria assessed via both spot urine and 24-hour urine collection without any major adverse events [91]. However, there is a clear need for future Phase 2/3 clinical trials regarding the efficiency and safety of cemdisiran therapy before reaching for a definitive conclusion, as the current state of knowledge is scarce.

To conclude, RNA-based therapies are a new field of pharmacotherapy, although currently in nephrology there are no approved drugs except for lumasiran in the treatment of primary hyperoxaluria. Nonetheless, we may expect to hear more about such novel agents in the upcoming years with potential clinical use.

#### Sibeprenlimab

APRIL, a member of tumor necrosis factor-alpha superfamily, has been implicated in the pathophysiology of IgA nephropathy through its actions on B cell-mediated immune response including the production of galactose-deficient IgA1, a key component in IgA nephropathy [92]. Sibeprenlimab, a humanized monoclonal antibody that binds and neutralizes the activity of APRIL, has shown to effectively suppress serum levels of total IgA and galactose-deficient IgA1 in preclinical studies and a Phase 1 clinical trial [93, 94]. A multicenter Phase 2 randomized double-blind placebo-controlled clinical trial has investigated the role of sibeprenlimab therapy in 155 biopsy-proven IgA nephropathy patients classified as high risk for progression after standard of care treatment. Sibeprenlimab therapy has led to a statistically significant decline in 24-hour protein-to-creatinine ratio compared with placebo at all doses after 12 months of therapy [95]. A Phase 3 clinical trial is also being conducted (NCT05248646) that may support regulatory approval by agencies for clinical use. Moreover, another monoclonal antibody therapy targeting the APRIL molecule, namely zigakibart, has shown promising outcomes in Phase 1 and 2 clinical trials [96] along with an ongoing Phase 3 trial (NCT05852938).

Another novel approach for IgA nephropathy is the use of a fusion protein capable of dual anti-B-cell Activation Factor (BAFF)-APRIL inhibition: namely atacicept, telitacicept, or povetacicept. A Phase 2b randomized double-blind clinical trial in 116 biopsy-proven IgA nephropathy patients has illustrated beneficial effects of atacicept therapy compared with placebo over 36 weeks of treatment in terms of proteinuria reduction and eGFR stabilization without any major adverse effects [97]. Furthermore, there are multiple ongoing clinical trials evaluating atacicept therapy among IgA nephropathy patients (NCT04716231, NCT07020923, NCT06983028). Similarly, a Phase 2 randomized placebo-controlled clinical trial in 44 IgA nephropathy patients with eGFR >35 ml/min/1.73 m^2^ and proteinuria ≥750 mg/day after receiving standard of care therapy has illustrated a considerable decline in proteinuria over a 24-hour clinical trial period [98]. Moreover, Phase 3 clinical trials are underway (NCT05596708, NCT06654596, NCT07052981). On the other hand, there are currently no published clinical trials regarding the effectiveness of povetacicept therapy on such a patient population in an ongoing Phase 3 clinical trial (NCT06564142).

To conclude, the APRIL molecule appears to surpass other pathophysiological targets in IgA nephropathy as a therapeutic target with multiple agents with single or dual inhibitory activity, and has been shown to elicit significant proteinuria decline. However, there is currently a lack of data regarding the long-term safety of such APRIL inhibitors along with potential concerns regarding infectious complication risk associated with mild drug-induced hypogammaglobulinemia and formation of anti-drug antibodies hypothetically reducing the activity of those medications. Nevertheless, APRIL inhibitors are expected to receive agency approvals for clinical use and potentially make a name for themselves.

#### Baxdrostat

As the renin–angiotensin–aldosterone system plays a central role in the pathogenesis of hypertension in both general population and CKD patients, alternative approaches targeting this mechanism offer potential improvements in blood pressure control. Aldosterone synthase inhibitors including baxdrostat offer decline in aldosterone synthesis without leading to an undesired increase in cortisol production [99, 100]. A recent multicenter double-blind placebo-controlled Phase 2 randomized controlled clinical trial has evaluated the efficacy and safety of baxdrostat therapy on 195 CKD patients with uncontrolled blood pressure, defined as systolic blood pressure ≥140 mmHg unless diabetes mellitus is present, under maximally tolerated doses of ACEi or ARB therapy and with eGFR of 25–75 ml/min per 1.73 m^2^ and UACR ≥100 mg/g creatinine. Baxdrostat therapy over a 26-week trial period has led to a statistically significant decline in systolic blood pressure, with a mean placebo-corrected decline of −8.1 mmHg (95% CI −13.4 to −2.8 mmHg, *P* = .003). Such improved systolic blood pressure control was also accompanied by significant improvements in diastolic blood pressure and albuminuria. However, 41% of the participants on the baxdrostat arm experienced hyperkalemia [101], potentially highlighting the need for future large-scale clinical trials in this field exploring safety concerns.

#### Cotadutide

Cotadutide, a novel dual agonist agent acting on GLP-1 and glucagon receptors, leads to upregulated insulin release, induction of satiety, and slowing of gastric emptying on its actions through GLP-1 receptor and increase in energy expenditure and decline in liver steatosis on its actions on glucagon receptors [102]. The role of cotadutide therapy on kidney function has recently been investigated in clinical trials along with its beneficial effects on weight and glycemic control. A Phase 2b placebo-controlled randomized clinical trial has investigated the effects of cotadutide therapy among 248 type II diabetes patients and CKD with eGFR between 20 and 90 ml/min/1.73 m^2^ and UACR >50 mg/g creatinine over a 26-week period. Cotadutide therapy has led to statistically significant and dose-dependent decline in UACR (−43.9%, 95% CI −54.7 to −30.6 with a 300 μg dose; −49.9%, 95% CI −59.3 to −38.4 with a 600 μg dose) [103]. Similarly, benefits of cotadutide therapy for albuminuria were demonstrated in another Phase 2a randomized clinical trial involving type II diabetes mellitus patients with baseline eGFR of 30–59 ml/min/1.73 m^2^ [104]. Moreover, the pharmacokinetic profile analysis of cotadutide therapy has revealed that cotadutide therapy is safe and tolerable among patients with kidney dysfunction without a need for dosage adjustment [105].

The significant decline in albuminuria in a diabetic patient population heavily treated with standard of care including RAAS blockers and SGLT-2 inhibitors is promising. However, there is a clear need for future large-scale clinical trials with longer follow-up periods for the better establishment of effects of cotadutide therapy on kidney outcomes.

### Future of kidney replacement therapies

Kidney replacement therapy (KRT) remains the cornerstone of life-sustaining treatment for patients with ESKD, and is currently delivered almost exclusively through dialysis modalities or kidney transplantation. The global demand for KRT continues to increase; it is estimated that ∼4.6 million individuals worldwide are living with kidney failure requiring dialysis or transplant: a figure that has risen steadily over recent decades [106]. This escalating burden is largely attributable to aging populations and the growing prevalence of diabetes mellitus and hypertension, the leading causes of ESKD [106]. While these modalities are lifesaving, they are resource-intensive, impose a substantial burden on patient quality of life, and fail to fully reproduce the complex homeostatic, metabolic, and endocrine functions of native kidneys. These limitations have stimulated an intensive search for innovative KRT paradigms.

**Table 1:** The potential perspectives in the future of nephrology with their advantages and disadvantages.

Biomarker	Advantages	Disadvantages	Clinical readiness
Calprotectin	✓ Reliable and non-invasive tool to differentiate pre-renal causes of AKI from intrinsic AKI ✓ Earlier detection of AKI compared to traditional biomarkers such as creatinine and cystatin C ✓ Reliable prediction of AKI-related clinical outcome including the risk for progression to KRT	Lack of well-established cutoff values for diagnostics Low number of clinical trials with limited number of patients	Potential tool for the differentiation of intrinsic AKI from pre-renal AKI along with earlier detection Requires future large-scale prospective clinical trials prior to widespread clinical use Potentially may become available in the upcoming years in clinical practice
suPAR	✓ Earlier detection of AKI compared to traditional biomarkers such as creatinine and cystatin C ✓ Not affected by confounding factors such as age, gender, muscle mass, or nutritional status ✓ Reliable prediction of future eGFR decline among patients presenting with kidney injury but yet with normal eGFR	Lack of well-established cutoff values for diagnostics Low number of clinical trials with limited number of patients	Potential tool for earlier detection of kidney injury with elimination of non-renal confounding factors Potentially may become available in the upcoming decades in clinical practice
CXCL9	✓ Reliable prediction of immune-checkpoint inhibitor-associated interstitial nephritis episodes and allograft rejection episodes among kidney transplant recipients	Lack of clinical data except for ICI therapy and kidney transplant recipients Lack of well-established cutoff values for diagnostics Low number of clinical trials with limited number of patients	Potential tool for the diagnosis of immune-checkpoint inhibitor-associated interstitial nephritis and allograft rejection episodes Potentially may become available in the upcoming decades in clinical practice
KRTs			
WAK	✓ Higher patient compliance and achievement of more physiological state mimicking kidneys ✓ Favorable hemodynamic and metabolic tolerance	Low number of clinical trials with limited number of patients	Potentially may become available in the upcoming decades in clinical practice
Mesenchymal stem cells	✓ Potentially better preservation of residual kidney function among CKD patients with safe adverse effect profile	Data in the clinical use of mesenchymal stem cells in the field of nephrology is preliminary and requires future large-scale clinical trials to prove efficacy	At proof-of-concept state and far from being clinically available
Kidney transplantation			
cfDNA	✓ The diagnosis of antibody-mediated or cellular rejection episodes without the hemorrhagic and infectious complication risks induced by allograft kidney biopsy Potentially earlier diagnosis of rejection episodes without well-established histopathological signs of rejection Follow-up of anti-rejection treatment response	Low number of clinical trials with limited number of patients Lack of well-established cutoff values for the diagnosis of rejection episodes	Clinically available tool for the diagnosis of allograft rejection episodes potentially prior to the establishment of histopathological alterations
Xenotransplantation	✓ A potential to overcome ‘”organ shortage” in the kidney allocation system for ESKD patients	Lack of clinical data regarding the efficacy and safety of such an approach Potentially high risk for allograft failure due to xenoantigens	At proof-of-concept state and far from being clinically available
MDR-101	✓ Aiming for an “immune-tolerant” state with no need for maintenance immunosuppressive regimen potentially reducing infectious and malignant complications	Potential risks with conditioning regimens including bone marrow suppression, mucositis and infectious complications Potential risk for loss of chimerism or so-called immune-tolerant state over time Potential risk for graft-versus-host disease Need for chimerism follow-up	Potentially may become available in the upcoming years in clinical practice

#### Advances in dialysis delivery and personalization

Over the last decade, substantial advances have been made in the delivery of dialysis, with a particular focus on enhancing patient autonomy, safety, and individualization of care. New-generation dialysis systems have become more compact, user-friendly, and technically sophisticated, facilitating wider adoption of home hemodialysis. These devices frequently incorporate touch-screen interfaces with stepwise guidance and integrated troubleshooting, significantly reducing training time and lowering the barrier for home use [107]. In addition, many systems now integrate on-board water purification and on-demand dialysate generation, eliminating the need for separate water treatment units or the storage of large dialysate volumes, as the device can be connected directly to a domestic tap and drain [107]. Continuous monitoring by multiple sensors with Wi-Fi connectivity enables remote supervision by dialysis centers, potentially improving safety and adherence among patients dialyzing at home. Such developments, exemplified by devices such as the Tablo system, have been associated with streamlined dialysis delivery and improved clinical outcomes [107].

In parallel, the conventional “one-size-fits-all” prescription of thrice-weekly hemodialysis is increasingly being questioned. An emerging concept is incremental hemodialysis, in which patients with substantial residual kidney function commence dialysis with fewer or shorter sessions and the dose is escalated only as native kidney function declines. Accumulating data indicate that incremental-start strategies can preserve residual kidney function and achieve comparable survival and hospitalization outcomes relative to immediate full-dose dialysis [108].

A broader goal is the personalization of dialysis dosing by adjusting treatment frequency, duration, and ultrafiltration rates according to an individual’s volume status, toxin generation, and comorbidity profile. Digital health tools and artificial intelligence have the potential to assist in tailoring dialysis prescriptions, integrating real-time physiologic and laboratory data. In the ICU, modern CRRT platforms already incorporate feedback algorithms that automatically adjust treatment parameters to maintain the prescribed dose, thereby narrowing the gap between prescribed and delivered clearance [109]. Similar data-driven decision-support systems in the chronic dialysis setting may optimize solute removal and fluid management on a patient-specific basis [109]. Overall, these developments reflect a shift toward variable KRT regimens that adapt dynamically to patient-specific conditions rather than rigid protocols, with the aim of improving tolerability, preserving residual kidney function, and enhancing outcomes.

#### Wearable artificial kidneys

Long-standing aspiration in the field of nephrology is the development of a wearable artificial kidney (WAK), a miniaturized device capable of providing continuous dialysis and thereby mimicking the continuous function of native kidneys. Such a device has the potential to liberate patients from fixed dialysis schedules and the physical confines of dialysis units, while attenuating the peaks and troughs in solute and volume status associated with intermittent hemodialysis. The essential components of a WAK include miniaturized blood and dialysate pumps, highly efficient sorbent-based dialysate regeneration systems, biocompatible membranes, portable power sources, and integrated monitoring and safety systems [110].

Several peritoneal- and hemodialysis-based wearable systems are currently at various preclinical and clinical stages of development, with differing technical configurations and degrees of clinical validation [110]. Early pioneering work from the Vicenza group led to the ViWAK system, a peritoneal dialysis (PD)-based WAK using a double-lumen PD catheter, polystyrenic resin, and activated carbon with conventional glucose dialysate. ViWAK demonstrated proof-of-concept *in vitro*, showing feasible sorbent-based dialysate regeneration, but did not progress to clinical testing [111].

Subsequent efforts have focused on single-lumen, sorbent-assisted PD systems that continuously regenerate a small volume of dialysate. The Automated WAK (AWAK) utilizes a single-lumen PD catheter and a modified REDY sorbent cartridge to enable tidal PD with continuous dialysate regeneration, resulting in a lightweight “waterless” device [112, 113]. In the first-in-human safety study, 15 ESKD patients underwent >100 AWAK sessions without any device-related serious adverse events and with acceptable small-solute clearances, supporting the feasibility of ambulatory use [114]. Based on these encouraging findings, pre-pivotal trials and home-use studies are ongoing, and AWAK has received FDA Breakthrough Device Designation, highlighting its potential to transform PD delivery in the outpatient setting [114].

The WEAKID (WEarable Artificial KIDney) platform builds on a similar conceptual framework, employing a single-lumen PD catheter and an external sorption unit containing ion exchangers and activated carbon to support continuous-flow PD. In uremic pig models, WEAKID-based continuous-flow PD improved small-solute clearance while maintaining hemodynamic stability [115]. A first-in-human clinical trial has recently been initiated to evaluate the safety, efficacy, and usability of WEAKID in ESKD patients, representing a critical step toward clinical translation (NCT06314503).

The Carry Life System (CLS) is another continuous-flow PD system that utilizes two single-lumen PD catheters and replaceable sorbent cartridges. Early feasibility data suggest that CLS can achieve adequate solute clearance with substantially reduced dialysate volumes compared with conventional PD, and enrollment into larger clinical trials is ongoing [116, 117].

In parallel, wearable hemodialysis devices employing a double-lumen vascular catheter and compact sorbent-based dialysate regeneration loops have completed short-term pilot trials. These systems have demonstrated acceptable urea and creatinine clearance and favorable hemodynamic tolerance in 24-hour studies; however, obstacles such as reliable anticoagulation, further miniaturization of pumps and sensors, and prevention of carbon dioxide bubble formation in the dialysate currently limit longer-term use [118].

Collectively, these PD- and HD-based WAK prototypes illustrate rapid progress toward truly portable and potentially wearable KRT. Nevertheless, substantial engineering refinement, robust longer-term safety and performance data, and demonstration of cost-effectiveness will be required before these technologies can be widely adopted in routine clinical practice.

#### Stem cell-based therapies and regenerative medicine

Stem cell-based and regenerative approaches represent another rapidly evolving area in nephrology, with the overarching goal of restoring rather than merely replacing kidney function. Mesenchymal stem/stromal cells (MSCs), which are multipotent cells derived from sources such as bone marrow, umbilical cord, and urine, have been extensively evaluated in preclinical models. However, their primary renoprotective effects appear to be mediated through paracrine mechanisms, including the secretion of growth factors and extracellular vesicles that modulate immune responses, attenuate inflammation, inhibit apoptosis, and promote tissue repair. In animal models, MSC therapy has been shown to ameliorate toxic and ischemic AKI, diabetic nephropathy, and progressive CKD via mechanisms such as upregulation of autophagy, reduction of fibrosis, and protection against oxidative stress. MSC-derived extracellular vesicles, in particular, have been demonstrated to prevent vascular calcifications in CKD and reduce fibrosis in obstructive nephropathy models [119]. Similarly, MSC infusions have activated anti-apoptotic and anti-inflammatory pathways in models of rhabdomyolysis-associated AKI and lupus nephritis, leading to improved outcomes [120].

Translation of these promising experimental data into human disease has been gradual but is now gaining momentum. Early-phase clinical studies suggest that MSC infusions are generally safe in CKD patients and provide preliminary signals of efficacy. The NEPHSTROM trial, a randomized controlled study conducted across Europe in patients with type II diabetes and progressive CKD, evaluated a single intravenous dose of allogeneic bone-marrow-derived MSCs (ORBCEL-M). At 18 months of follow-up, MSC-treated patients demonstrated better preservation of kidney function compared with placebo, with an acceptable safety profile [121].

A smaller study published in 2025 investigated intra-arterial delivery of autologous MSCs in patients with diabetic CKD. In this trial, MSC-treated participants experienced a slower rate of eGFR decline over the subsequent year than would have been anticipated based on historical progression rates, without significant adverse events [122]. Also, there is an ongoing clinical trial evaluating such a therapeutic approach on 53 participants [123]. Although these clinical data remain preliminary, they support MSC-based therapies as a potentially valuable adjunct to conventional renoprotective strategies, with the long-term goal of delaying progression to dialysis-dependent kidney failure.

Chimeric antigen receptor-T cell (CAR-T) approaches are also being actively investigated and appear very promising for refractory forms of systemic lupus erythematosus, ANCA-associated vasculitis, membranous nephropathy, and monoclonal gammopathy-related nephropathies [124].

#### Continuous KRT and multiorgan support in critical care

In the intensive care unit, continuous KRTs (CRRTs) have become a fundamental modality for the management of AKI in hemodynamically unstable patients, given its superior hemodynamic tolerability compared with intermittent hemodialysis [109]. Over the past decade, advances in CRRT technology have broadened its applications and facilitated the emergence of integrated multiorgan support concepts. Modern CRRT platforms are increasingly designed not only to provide renal support but also to interface with systems used for other failing organs.

For instance, some CRRT machines can perform therapeutic plasma exchange by incorporating dedicated plasma filters into the circuit, thereby removing the need for separate plasmapheresis devices [109]. CRRT systems can also be coupled with extracorporeal CO₂ removal (ECCO₂R) devices in patients with refractory respiratory failure, enabling simultaneous control of solute and fluid balance and partial respiratory support [125]. In the context of extracorporeal membrane oxygenation for combined cardiac and respiratory failure, CRRT has been successfully integrated into the extracorporeal membrane oxygenation circuit, allowing a single platform to provide multiorgan support in the most critically ill patients (35 998 214). This sequential multiorgan support paradigm brings together kidney, lung, and circulatory support in a unified extracorporeal system and represents an important step toward “one-stop” critical care technologies [125].

Another rapidly developing area is the use of novel blood purification strategies targeting sepsis and cytokine storm syndromes. High cutoff membranes and adsorptive cartridges have been designed to remove inflammatory mediators, bacterial toxins, and other pathogenic molecules from the circulation [126]. These hemoadsorption devices, when incorporated into CRRT circuits, can bind cytokines such as interleukin-6 directly from blood, with the intent of modulating the host inflammatory response in septic shock [126]. Although multiple studies have demonstrated reductions in circulating cytokine levels, the impact of these interventions on hard clinical endpoints such as mortality remains uncertain, and current evidence is largely derived from small, heterogeneous cohorts [126].

Finally, there is increasing interest in leveraging advanced monitoring and control systems to optimize CRRT delivery. Widespread use of sensors and automation has already improved the accuracy of fluid removal and dosing. Ongoing research focuses on integrating predictive algorithms and artificial intelligence into CRRT platforms to guide real-time adjustments in therapy, such as tailoring ultrafiltration rates to a patient’s volume responsiveness or identifying the optimal timing for initiation and discontinuation of CRRT in AKI [109]. These data-driven approaches have the potential to refine KRT delivery, enhance kidney recovery and more effectively embed CRRT within complex multiorgan support strategies in the ICU.

### Artificial intelligence in nephrology

**Figure 1:**
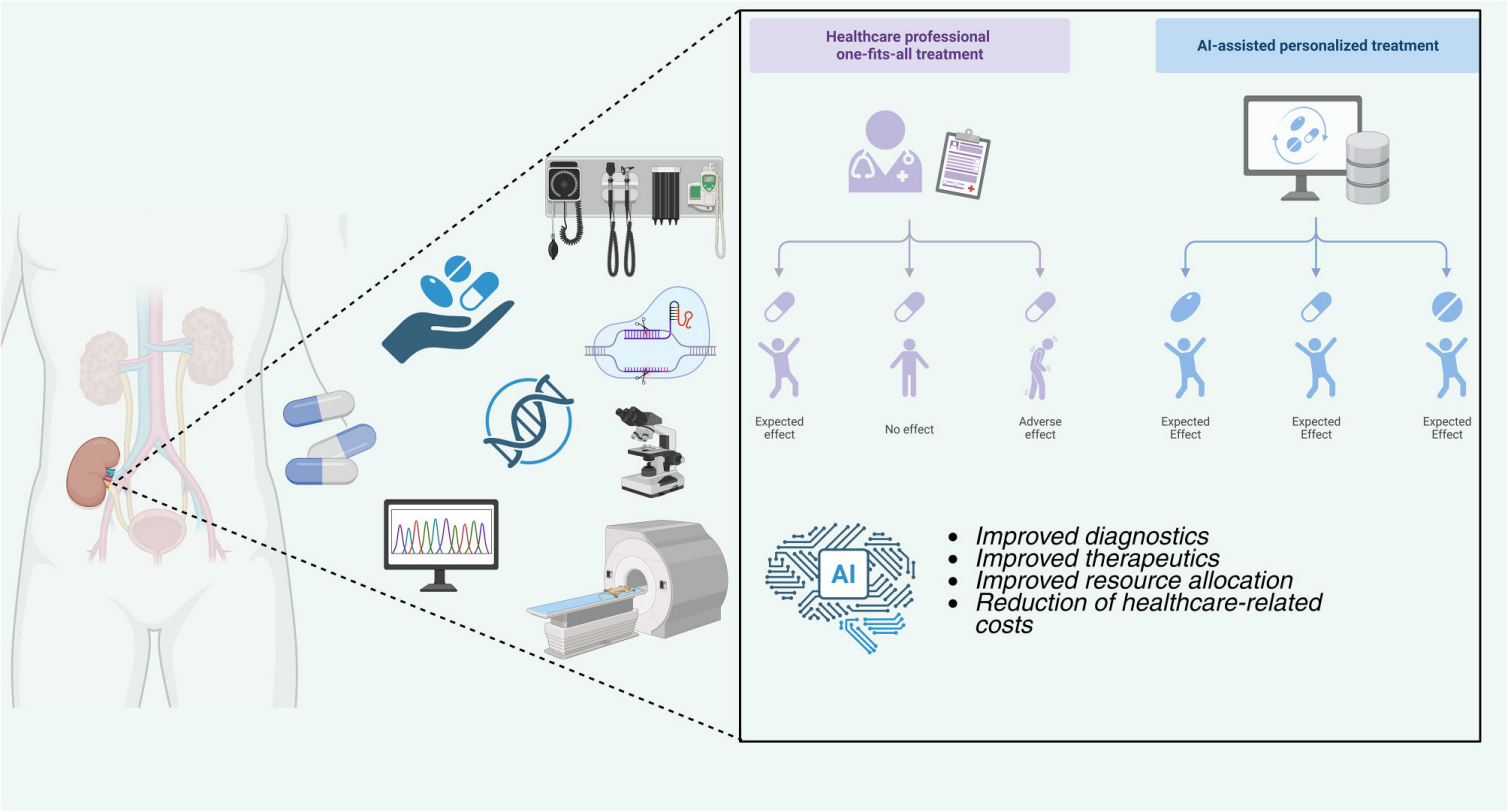
The potential role of artificial intelligence-based modalities in the field of nephrology. The integration of AI in nephrology may lead to improvements in diagnostics and therapeutic perspectives, better resource allocation, and decline in healthcare-related medical expenses.

Artificial intelligence (AI) and machine learning (ML) are increasingly being integrated into nearly every aspect of nephrology, spanning early disease detection, advanced imaging, dialysis prescription, transplantation, and critical care. Rather than a single method, AI comprises a diverse set of algorithms capable of analyzing high-dimensional datasets that exceed human analytic capacity. It has previously been emphasized that nephrology is particularly well positioned for AI-driven transformation, owing to the highly data-intensive nature of CKD management, dialysis, and transplantation (Figure 1) [127].

#### Risk prediction, early detection, and biomarker integration

Traditional CKD risk stratification relies primarily on eGFR, albuminuria, and a limited set of comorbidities. Multiple ML models trained on large electronic health record (EHR) datasets have now demonstrated superior performance over conventional regression-based equations for predicting incident CKD, rapid eGFR decline, or kidney failure, especially in intermediate-risk patients. These models typically combine demographics, longitudinal laboratory trajectories, comorbidity burden, and medication data, and can be recalibrated to local practice patterns. Similarly, AI frameworks have been developed to predict AKI in hospitalized or critically ill patients hours before creatinine rises, using continuous vital signs, medication exposures, and laboratory trends [128].

An important future direction is the incorporation of novel biomarkers into these models. Circulating and urinary markers such as calprotectin, suPAR, CXCL9 can be integrated with routine clinical and EHR data to generate “digital biomarker” signatures that detect kidney injury earlier and more specifically than any single analyte. As more prospective cohorts collect both advanced biomarkers and granular EHR data, AI-based multimarker risk scores are likely to complement or replace current one- or two-marker thresholds for triggering further diagnostic workup or nephrology referral [129].

#### Optimization of dialysis and continuous RRT

In chronic hemodialysis, AI has been explored for predicting intradialytic hypotension (IDH) and other complications. Multiple ML and deep learning models using pre-dialysis or real-time treatment data have achieved high accuracy for IDH prediction, enabling early adjustment of ultrafiltration rates, dialysate sodium, or treatment time [130]. Recently, a prospective implementation of an AI-driven IDH prediction system demonstrated clinically meaningful reductions in hypotensive events and suggested health-economic benefits, highlighting the potential of AI as real-time decision support in dialysis units [130].

AI is also being applied to personalize dialysis prescriptions more broadly. Models trained on large dialysis datasets can forecast hemoglobin trajectories and recommend erythropoiesis-stimulating agent dosing, predict hospitalization risk, or individualize target dry weight and ultrafiltration profiles. In the ICU, where continuous RRT is frequently integrated with other extracorporeal therapies, AI-assisted control systems are being investigated to optimize delivered CRRT dose, minimize fluid imbalance, prolong filter life, and support standardized multiorgan support protocols [131].

#### AI in kidney transplantation and organ allocation

Across the transplant pathway, AI is being used to enhance donor-recipient matching, predict graft and patient outcomes, and improve allocation efficiency. Interpretable ML models trained on national registry data can outperform traditional scores in predicting graft survival and patient mortality after kidney transplantation [132]. Dedicated AI tools such as the Live-Donor Kidney Transplant Outcome Prediction (L-TOP) model for living donation or similar models for deceased donors provide individualized risk estimates that may support informed consent and organ acceptance decisions [132].

Beyond outcome prediction, AI is increasingly applied to the allocation process itself. Optimization algorithms that incorporate predicted graft longevity and recipient survival have been proposed to reduce discard rates and better align organ quality with recipient risk [133]. In parallel, ML analysis of hypothermic machine perfusion data, cfDNA levels, and donor-specific antibody profiles may refine non-invasive detection of subclinical injury and guide intensity of immunosuppression and biopsy strategies after transplantation [133].

#### Application to clinical practice

Even though AI-based modalities offer promising results in small number of early retrospective clinical studies, the evidence is far from achieving clinical use. Despite impressive technical performance, most AI applications in nephrology remain at the proof-of-concept stage. Common limitations include training on retrospective, single-center datasets with limited external validation, uncertain generalizability across healthcare systems, and vulnerability to dataset shift over time [134]. There is a clear need for future prospective large-scale clinical validation studies before widespread clinical adoption. Additional major concerns while implementing AI-based modalities in nephrology, not distinct from any other medical specialty, include ethical and legal concerns. Ethical and legal concerns that required consideration and addressing with the evolving use of AI-based modalities include: (i) issues of accountability and liability when AI possibly provides an improper diagnosis or therapeutic plan; (ii) data privacy and confidentiality referring to the large-scale clinical data while designing and training AI modalities; (iii) potentially widening the gap between patients based on socioeconomic status, as not all patient groups may have similar access to AI modalities; and (iv) unclear methodologies of AI modalities not providing transparent methodology utilized in the decision-making process. Along with the improvements in clinical evidence and prospective clinical validation of AI-based modalities in nephrology, there is a definitive need for legal regulations by authorities.

### Future perspectives for kidney transplantation

Kidney transplantation, the current gold standard therapeutic option for suitable ESKD patients, offers superior survival and quality of life advantage over other KRT, however, certain challenges including acute and/or chronic rejection, immunosuppression-related adverse events, graft failure, organ shortages, and immunological barriers should not be overlooked. With the implementation of novel therapies including advancements in desensitization procedures, complement inhibitors such as eculizumab and ravulizumab, anti-CD20-based therapies such as obinutuzumab, and plasma cell-directed therapies such as daratumumab, greater access to kidney transplantation with higher success rates has been achieved. Nevertheless, there is a clear need for future advancements in the field of kidney transplantation to reach higher success rates.

#### MDR-101

**Figure 2:**
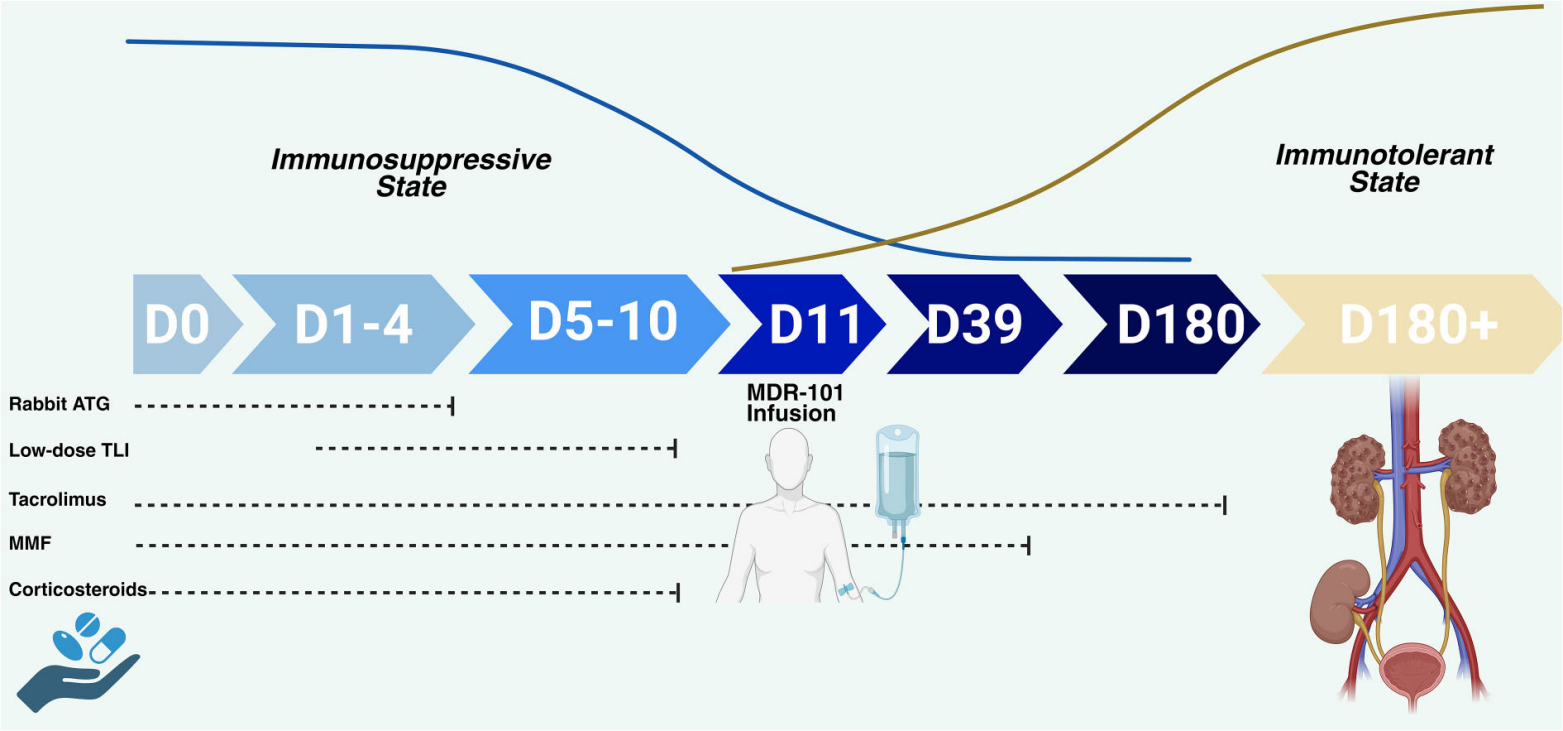
The utilization of MDR-101 in the kidney transplantation process. MDR-101 treatment is infused to kidney transplant recipients at post-transplant day 11 following a non-myeloablative conditioning regimen including rabbit anti-thymocyte globulin and low-dose total body irradiation. The maintenance immunosuppressive regimen is withdrawn gradually with tapering and discontinuation of corticosteroids on post-transplant day 10, mycophenolate mofetil on day 39 and tacrolimus on day 180. Abbreviation: MMF, mycophenolate mofetil.

MDR-101 is a cellular therapy derived from the living donor’s peripheral blood and includes primarily CD34 positive hematopoietic stem cells, CD3 positive T cells, and progenitor cells. The primary aim of such an approach is to induce an “immune-tolerant” state between two-haplotype HLA-matched living donor kidney transplants to achieve a post-transplant state with no need for maintenance immunosuppression. The hypothetically achieved immune-tolerant state may not only lower the risk for rejection or graft failure rates, but also prevent immunosuppression-related adverse events including drug toxicities or infectious complications. It is administered at post-transplant day 11 after a non-myeloablative conditioning regimen as in autologous hematopoietic stem cell transplantation. A Phase 3 randomized placebo-controlled clinical trial has utilized rabbit anti-thymocyte globulin and low-dose total lymphoid irradiation as the conditioning regimen followed by MDR-101 administration at post-transplant day 11. Corticosteroid therapy was withdrawn on day 10 and mycophenolate at day 39 whereas tacrolimus was tapered starting on day 180 (Figure 2). Out of 20 patients receiving MDR-101 infusion, 16 have achieved the primary endpoint of the trial, which is immunosuppressive-free follow-up over 2 years without any episode of donor-specific antibody formation, graft-versus-host disease, or mortality. The re-initiation of immunosuppressive regimens in four patients was due to recurrent IgA nephropathy (*n* = 1), rejection (*n* = 1), rejection plus recurrent IgA nephropathy (*n* = 1), and borderline biopsy findings (*n* = 1) [135]. Even though no major adverse events had been reported in this Phase 3 clinical trial with MDR-101 therapy, the potential concerns for certain events continue and include: (i) conditioning regimen-related toxicities including cytopenia, mucositis, and infectious complications; (ii) a risk of loss of chimerism or failure to achieve an immune-tolerant state leading to rejection episodes following the immunosuppressive regimen taper; (iii) a requirement for chimerism follow-up; (iv) hypothetical concern for graft-versus-host disease with donor-derived T cells; and (v) hypothetical concerns of malignant conditions with early conditioning regimen. Even though the immunosuppressive-free regimen after kidney transplantation is intriguing and exciting with promising early results, there is an absolute need for future large-scale clinical trials with long-term follow-up periods.

#### Felzartamab

The benefits and promising outcomes of daratumumab in the management of antibody-mediated rejection episodes after kidney transplantation have led to further investigation of plasma cell-based therapies including felzartamab. Administration of such therapy to IgA nephropathy patients has led to promising outcomes in proteinuria reduction [136], which led to an investigation in kidney transplant recipients. A Phase 2 double-blind placebo-controlled clinical trial has revealed beneficial outcomes of felzartamab therapy in the management of antibody-mediated rejection, however, patients experiencing rejection episodes at least 180 days after transplantation were included in this trial [137]. Nevertheless, such clinical studies are too premature to draw definitive conclusions.

#### Cell-free DNA

The gold standard diagnostic approach for acute or chronic antibody-mediated or cellular rejection episodes is currently allograft biopsy, which is not an intervention without complications that include hemorrhagic and infectious complications. A prospective single-center clinical study of 108 allograft biopsy samples from 106 kidney transplant recipients has illustrated the predictive value of cell-free DNA (cfDNA) on rejection episodes, as patients with antibody-mediated rejection have the highest level of cfDNA, followed by T cell-mediated rejection, borderline rejection, and no rejection, respectively. Similarly, anti-rejection therapies have led to statistically significant declines in cfDNA levels at days 7, 30, and 90 after biopsy [138]. Even though Benning and colleagues have proposed cfDNA as a marker for rejection episodes similar to donor-specific antibody titers and it may aid the decision-making process in treatment response evaluation or diagnostic procedure, it is too premature to reach for such a strong statement. Similar outcomes have also been established in another multicenter prospective cohort study of 300 biopsy samples from 289 kidney transplant recipients [139]. The integration of cfDNA measurements in decision-making processes has recently been evaluated in a single-center randomized clinical trial. A group of 40 kidney transplant recipients with high DSA titers and eGFR ≥20 ml/min/1.73 m^2^ without any previous biopsy-proven rejection episodes were randomized to undergo allograft biopsy depending on cfDNA or clinician-guided decisions. This intervention led to a median of 2.8 months earlier diagnosis of antibody-mediated rejection episodes (*P* = .003) while positive and negative predictive value of cfDNA were calculated as 77% and 85%, respectively, when >50 copies/ml was utilized as the cutoff for biopsy [140]. Even though future clinical trials are required for a better understanding of such an issue, we believe cfDNA measurements may have high potential to be included in the evaluation of kidney transplant recipients with a suspicion for rejection, similar to donor-specific antibody titers, and may eliminate the need for performing unnecessary graft biopsies.

#### Xenotransplantation of kidneys

Xenotransplantation, the transplantation of organs from non-human species into humans, has re-emerged as a promising strategy to alleviate the persistent organ shortage in kidney transplantation. Historically, clinical attempts at organ xenografting were uniformly unsuccessful, primarily due to immediate immune rejection, thrombotic complications, and the risk of cross-species pathogen transmission [141]. Advances in genetic engineering and cloning have now enabled the generation of genetically modified pigs whose organs are more compatible with the human immune system. Pigs are attractive donor candidates owing to their physiological similarity to humans, relatively short gestation and maturation times, and the feasibility of extensive genetic manipulation to reduce immunogenicity and xenozoonotic risk. Contemporary “designer pigs” typically harbor multiple gene edits, including knockout of key xenoantigens such as α-gal and the introduction of human complement-, coagulation-, and immune-regulatory genes [141].

In 2022, two high-profile experimental pig kidney xenotransplants in brain-dead human donors produced encouraging short-term results. In these studies, genetically modified pig kidneys transplanted into brain-dead recipients promptly produced urine with improved glomerular filtration and no evidence of hyperacute or early antibody-mediated rejection over approximately 54 hours of observation [142]. These proof-of-concept procedures demonstrated that hyperacute rejection can be prevented and that pig kidneys are capable of initiating function in a human physiologic environment.

In 2023, a gene-edited pig kidney was transplanted into a brain-dead man and maintained function for a record 61 days, representing the longest duration for which a xenogeneic solid organ has sustained human physiology to date [143]. Throughout the 2-month period, the xenograft produced urine, maintained electrolyte and fluid homeostasis, and obviated the need for dialysis. A mild rejection episode around 1 month was successfully managed by intensifying immunosuppression, after which the kidney continued to function. At explant, the organ appeared grossly normal and histologically viable [143]. Following the promising results from preclinical models of successful implementation and initial functioning of xenotransplantation of kidneys from gene-edited pig models, few cases were described for such an approach in clinical practice. Kawai and colleagues had transplanted a xenograft from a pig with 69 genetic edits including deletion of three glycan antigens, inactivation of porcine endogenous retroviruses, and insertion of seven human transgenes to a patient with exhaustion of all viable vascular access under a “single patient, expanded access authorization” approved by the Food and Drug Administration. The induction immunosuppressive regimen included ATG, rituximab, and tegoprubart, an Fc-modified aCD154 with addition of ravulizumab, an anti-C5 monoclonal antibody, based on high thrombotic microangiopathy risk whereas maintenance immunosuppressive regimen included tacrolimus, mycophenolic acid, and prednisone. A T cell-mediated rejection episode observed at post-transplant day 8 was successfully treated with ATG and pulse corticosteroid therapy with stabilization of serum creatinine at 1.5–2.2 mg/dl. The patient died at post-transplant day 52 with autopsy revealing signs of severe ischemic heart disease without any sign of rejection or thrombotic microangiopathy on the allograft [144]. The same group performed another xenotransplantation in 2025 on a 66-year-old male patient with diabetic ESKD with stable serum creatinine of 1.2–1.4 mg/dl at post-transplant month 5. Another study group, the NYU group, performed two xenotransplantations. One transplantation was performed on a patient on left ventricular assist device due to severe congestive heart failure with removal of xenograft on post-transplant day 47 due to insufficient blood flow, and the second transplantation was performed on a highly HLA-sensitized patient with stable xenograft function at an early post-transplant period with an irreversible rejection episode resulting in xenograft loss following a reduction in immunosuppressive regimen due to infectious complications [145].

These findings provide strong proof-of-principle that prolonged pig kidney function in humans is feasible and fuel optimism that clinical xenotransplantation trials in living patients may be achievable soon.

## CONCLUSION

**Figure 3:**
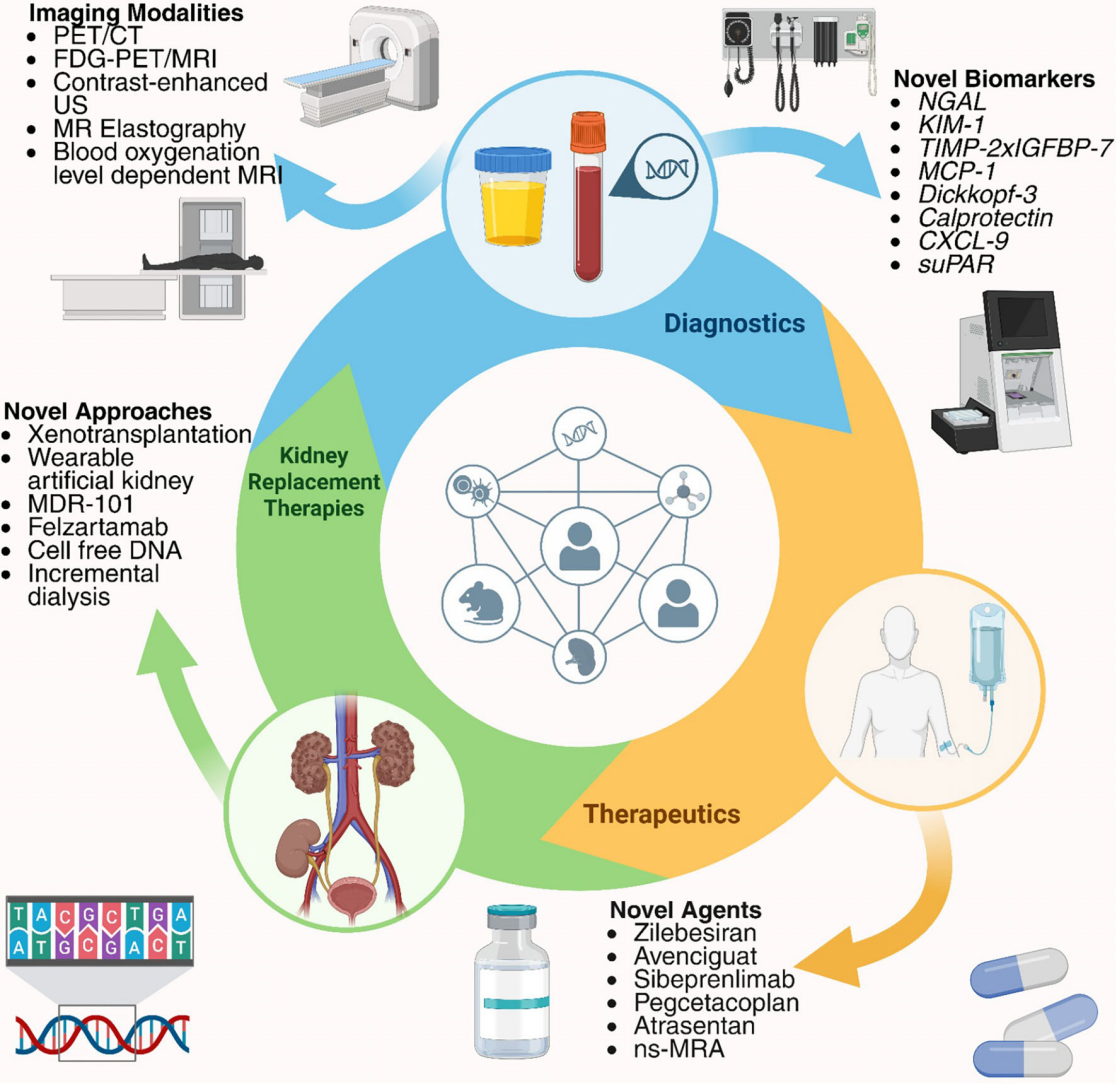
The advancements in the field of nephrology in terms of diagnostics, biomarker selection, and therapeutics. Abbreviations: US, ultrasound; ns-MRA, non-steroidal mineralocorticoid receptor antagonist; TIMP-2, urinary tissue inhibitor of metalloproteinases-2; IGFBP-7, insulin-like growth factor-binding protein-7; MCP-1, monocyte chemoattractant protein-1.

Nephrology is a rapidly growing field of medicine with promising preclinical and clinical data on novel diagnostic and therapeutic modalities (Table 1). Integration of novel diagnostic modalities into clinical practice may not only improve the sensitivity and specificity of diagnostic algorithms but also enable earlier diagnosis to prevent chronic irreversible damage at kidney parenchyma (Figure 3). Similarly, implementation of future therapeutic modalities developed via the integration of genetics, pharmacogenomics and pharmacodynamic approaches may lead to better prognosis for those kidney disorders.

## ETHICS STATEMENT

This review is based entirely on previously published literature. No new data involving humans or animals were collected. All sources are properly cited, and the authors declare the work is original, ethical, and free from conflicts of interest.

## ETHICAL APPROVAL

The study does not contain any studies with human participants or animals performed by any of the authors.

## Data Availability

No new data were generated in this paper. All authors approved the final version of the paper.
